# Atomic Charge Calculator III: a modern platform for calculating partial atomic charges

**DOI:** 10.1093/nar/gkag379

**Published:** 2026-04-27

**Authors:** Tomáš Raček, Martin Pilát, Ondřej Schindler, Gabriela Bučeková, Dominik Tichý, Karel Berka, Radka Svobodová

**Affiliations:** National Centre for Biomolecular Research, Faculty of Science, Masaryk University, Kamenice 5, Brno 625 00, Czech Republic; CEITEC - Central European Institute of Technology, Masaryk University, Kamenice 5, Brno 625 00, Czech Republic; Faculty of Informatics, Masaryk University, Botanická 68a, Brno 602 00, Czech Republic; National Centre for Biomolecular Research, Faculty of Science, Masaryk University, Kamenice 5, Brno 625 00, Czech Republic; CEITEC - Central European Institute of Technology, Masaryk University, Kamenice 5, Brno 625 00, Czech Republic; National Centre for Biomolecular Research, Faculty of Science, Masaryk University, Kamenice 5, Brno 625 00, Czech Republic; CEITEC - Central European Institute of Technology, Masaryk University, Kamenice 5, Brno 625 00, Czech Republic; Faculty of Informatics, Masaryk University, Botanická 68a, Brno 602 00, Czech Republic; Department of Physical Chemistry, Faculty of Science, Palacký University, 17. listopadu 12, Olomouc 771 46, Czech Republic; National Centre for Biomolecular Research, Faculty of Science, Masaryk University, Kamenice 5, Brno 625 00, Czech Republic; CEITEC - Central European Institute of Technology, Masaryk University, Kamenice 5, Brno 625 00, Czech Republic

## Abstract

Partial atomic charges provide a simplified description of the molecular electrostatic distribution that governs interactions with the surrounding environment. Owing to their widespread use in computational chemistry, chemoinformatics, and bioinformatics, fast and reasonably accurate empirical approaches remain highly relevant. Here, we introduce Atomic Charge Calculator III (ACC III), a web application for computing partial atomic charges for diverse molecular systems, including small organic molecules and proteins. ACC III builds on previous releases and implements 20 widely used empirical charge calculation methods, including the state-of-the-art SQE+qp approach with updated parameters. The platform integrates the modern Mol* viewer for interactive visualization and supports embedding computed charges directly into mmCIF files using a formally defined extension, enabling distribution of results in a community-standard, FAIR structural format. In addition to the web interface, ACC III provides a programmatic API for automated workflows and an enhanced command-line version for local deployment. The service is freely available at https://acc.biodata.ceitec.cz, with no login required, including for commercial use.

## Graphical Abstract

**Graphical Abstract:**
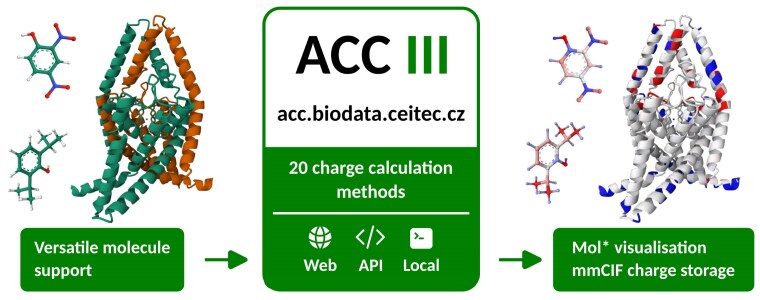

## Introduction

Partial atomic charges provide a simplified description of the electron distribution within a molecule and are widely used to model electrostatic interactions. Although they are not uniquely defined quantum-mechanical observables, they remain among the most practical and interpretable descriptors in computational chemistry. By approximating the redistribution of electron density caused by bonding and chemical environment, partial charges enable the modelling of intermolecular interactions and molecular recognition [1].

Because electrostatic interactions are long-range and often dominant, partial atomic charges play a central role in molecular mechanics force fields [2], molecular dynamics simulations [3], docking studies [4], and structure-based bioinformatics [5, 6]. They are also used to rationalize substituent effects [7], estimate physicochemical properties [8, 9], and parameterize atomistic models [2]. In large biomolecular systems, they frequently constitute the primary electrostatic term governing structural stability and interaction patterns.

Traditionally, partial atomic charges have been obtained exclusively through theoretical approaches, most commonly from quantum-mechanical calculations or from empirical and semi-empirical models derived from quantum data. Different computational schemes, either based on electron density partitioning [10, 11], electrostatic potential fitting [2], or electronegativity equalization [12], often yield numerically different values, reflecting the conceptual ambiguity of the quantity itself [1]. Recently, experimental approaches based on electron diffraction have demonstrated that partial charges can, under specific conditions, be inferred directly from crystallographic data [13]. However, such methods are currently limited to crystalline systems of sufficient quality and resolution and are not yet suited for routine large-scale computational workflows. For most applications, fast and robust computational approaches therefore remain indispensable. In particular, empirical approaches are well-suited for high-throughput applications and large-scale analyses, where deriving charges using quantum-chemical methods would be impractical, and are primarily intended for applications where strict compatibility with molecular mechanics force fields is not required.

Accessible web-based tools are particularly important in this context, as they lower the barrier to entry and enable charge calculations without specialized software installation or IT expertise. Atomic Charge Calculator I (ACC I) [14] addressed this need by providing an implementation of the Electronegativity Equalization Method [12] within a web interface, including strategies to handle large macromolecular structures efficiently. ACC II [5] substantially expanded this framework by incorporating 16 additional empirical methods, simplifying the user interface, and introducing improved visualization capabilities.

Subsequently, we developed specialized web applications focused on large structural repositories, namely PDBCharges [15] and AlphaCharges [16], which provide charges for structures from the Protein Data Bank (PDB) [17] and the AlphaFold Database [18], respectively. These tools are tailored to specific datasets but lack the universality of ACC.

Here, we present its third version (ACC III), the next stage in the Atomic Charge Calculator platform’s development. ACC III further extends the range of available empirical methods, improves interoperability through enhanced output formats and programmatic access, and introduces refined tooling for both interactive and automated workflows. Together, these improvements provide a mature tool for fast, empirical partial-charge calculations across diverse classes of molecules.

## Description of the web server

A central design goal of ACC II was to provide an intuitive interface that enables users to compute partial atomic charges with minimal configuration, including a one-click calculation option. After completion of the computation, a results page with interactive charge visualization is presented, together with the possibility to download the calculated charges in standard formats.

ACC III preserves this design philosophy while refining the user interface, improving responsiveness, and extending configuration options, such as enabling the calculation of multiple charge types for a single structure within a single workflow. The server accepts common structural input formats, including SDF (MOL V2000 and V3000 records), PDB, mmCIF, and Mol2. In addition to these incremental improvements, ACC III introduces several substantial enhancements, which are described in the following sections.

### Split-charge equilibration methods

While ACC II provided 17 empirical charge calculation methods, ACC III extends this set by three additional approaches based on the split-charge equilibration formalism. These include the original Split-charge Equilibration (SQE) [19], Split-charge Equilibration with initial formal charges (SQE+q0) [20], and Split-charge Equilibration with initial parameterized charges (SQE+qp) [6]. The full list of methods with implementation details and benchmarks is available at the ACC III website at https://acc.biodata.ceitec.cz/methods.pdf.

### Mol* integration and charge visualization

Visual representation of partial atomic charges is important for interpreting electrostatic features within a molecular structure. ACC III integrates Mol* [21], a modern molecular viewer that provides high performance and long-term support. Computed charges can be directly mapped onto the three-dimensional structure for interactive exploration.

In addition, we developed an official Mol* extension that enables visualization of partial atomic charges stored in mmCIF files using the custom categories defined by ACC III (see ‘Output charge formats’). This allows charge-annotated structures to be viewed in standard Mol* deployments outside the ACC III web interface.

### Optional user workspace

ACC III is fully accessible without any kind of registration. For users who wish to store and manage their data, optional authentication via Life Science Login [22] is available, allowing sign-in through their home institution without sharing credentials with the service. Authenticated users can manage input files, access previous calculations, and work within a persistent user workspace. Each registered account is allocated 500 MB of storage for submitted data and calculation results.

### Output charge formats

**Figure 1.**
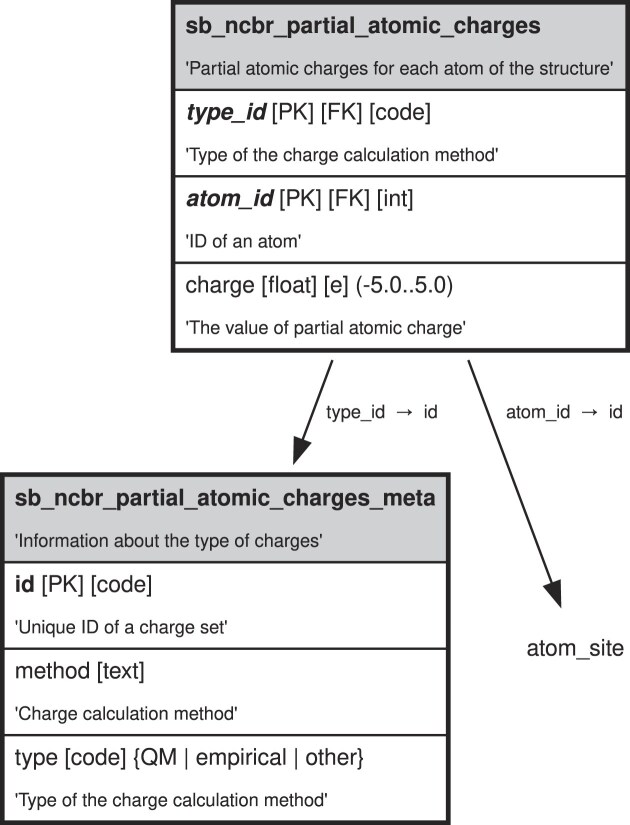
Schema of the mmCIF extension used for storing partial atomic charges in ACC III. The category sb_ncbr_partial_atomic_charges_meta defines individual charge sets (primary key: id), including the calculation method and type. The category sb_ncbr_partial_atomic_charges stores per-atom charge values (composite primary key: type_id, atom_id). Foreign key relationships link type_id to the metadata category and atom_id to atom_site.id, ensuring consistency with the structural definition.

ACC III provides charge output in the Mol2 format for small molecules, in the PQR format for protein-like molecules, and in plain text. However, these formats have inherent limitations. Therefore, ACC III also enables storing partial atomic charges directly within an mmCIF file using custom-defined categories: sb_ncbr_partial_atomic_charges_meta for metadata and sb_ncbr_partial_atomic_charges for the charge values themselves (Fig. 1). The data model is formally defined in an mmCIF extension dictionary (https://sb-ncbr.github.io/charges-schema/schemas/mmcif_charges_v10.dic), allowing standard mmCIF parsers to read the stored values.

### Interfaces

While the primary interface of ACC III is a web application, it also provides a web API to enable automated charge calculations. For more advanced use cases, such as computing charges for thousands of structures, the computational C++ core, ChargeFW2, can be installed and run locally. Python bindings are available to facilitate its use within the Python ecosystem. In addition, a Docker container is provided via Docker Hub (https://hub.docker.com/r/sbncbr/chargefw2) to simplify installation and deployment.

### Limitations

Since ACC III is not limited to a specific class of molecules, it does not automatically protonate input structures. Protonation strongly depends on molecular context and chemical environment, and a reliable general solution is not feasible for arbitrary molecular systems. Therefore, users must provide correctly protonated structures prior to charge calculation. While specialized tools (such as Hydride [23] and PDB2PQR [24]) can address protonation for well-defined molecule classes, such assumptions cannot be safely generalized in ACC III.

To ensure stable and fair access for all users, the public web API is subject to reasonable usage limits. Users requiring large-scale calculations are encouraged to use ChargeFW2 locally.

## Results and discussions

To demonstrate possible uses of the ACC III, we present two use examples. The interactive versions of these examples are available on the ACC III webpage. A third example on the ACC III webpage, illustrating dissociating hydrogens in phenols, was already described in the ACC II paper [5].

### Pore complex

**Figure 2.**
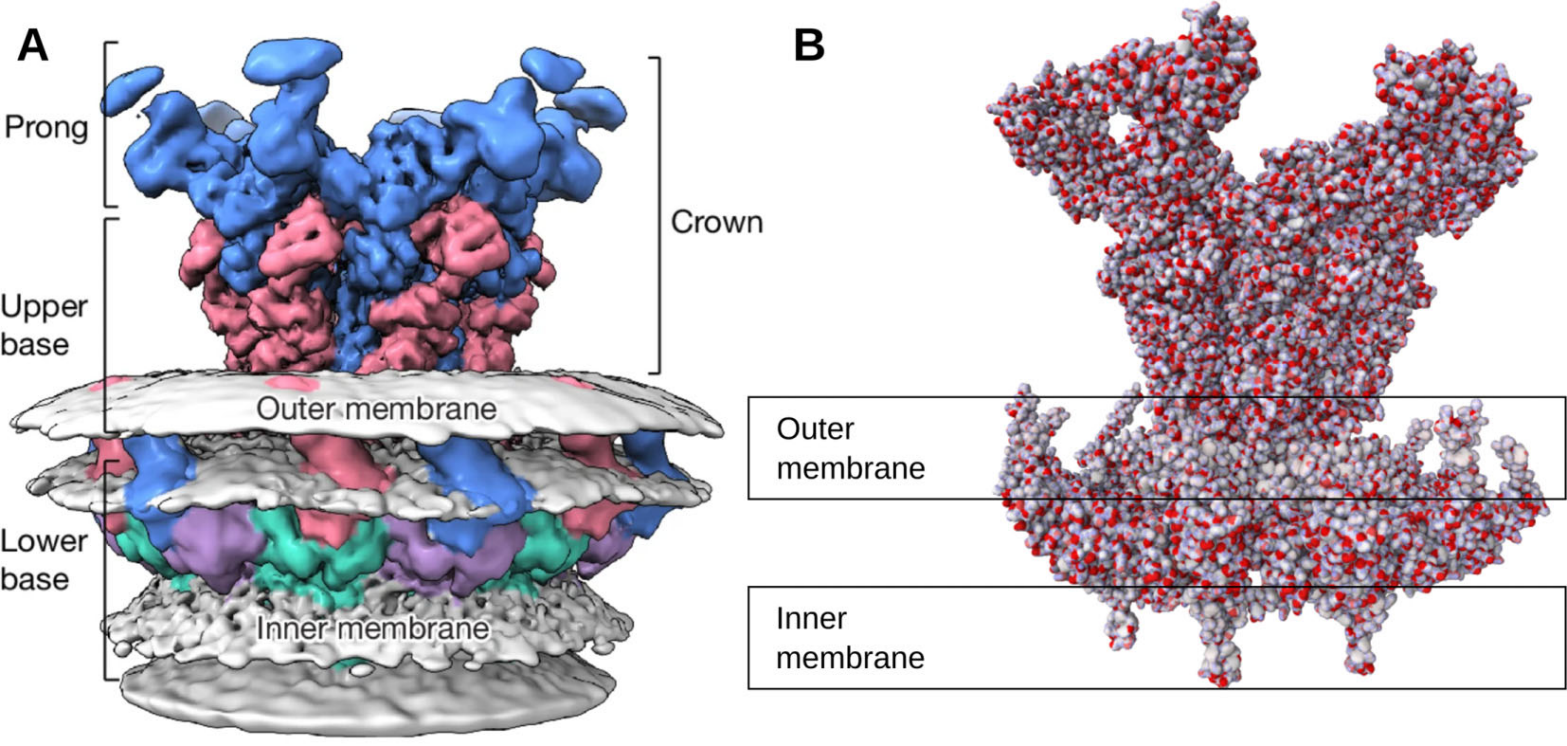
The SARS-CoV-2 pore (PDB ID *8yax*): (**A**) The pore penetrating the phospholipid bilayer of the intracellular host membrane (image taken from [25]). (**B**) Visualization of surface partial charges highlights the difference between the nonpolar transmembrane regions (mostly white due to near-zero charge) and the polar surfaces of the extracellular and cytoplasmic regions, which exhibit a heterogeneous distribution of positive (blue) and negative (red) charges.

The first example presents the fully hydrogenated SARS-CoV-2 pore complex (PDB ID *8yax*), resolved by cryogenic electron tomography [25]. Coronaviruses form these pores during replication to penetrate the phospholipid bilayers of intracellular host membranes (see Fig. 2 A) and transport viral RNA. The charge distribution clearly indicates the positions of the outer and inner membranes (see Fig. 2 B). Since both membranes are nonpolar, they exhibit a markedly higher occurrence of regions with partial atomic charges close to zero (colored white) and a lower proportion of charged regions (colored red and blue).

### Neuraminidase in complex with oseltamivir

**Figure 3.**
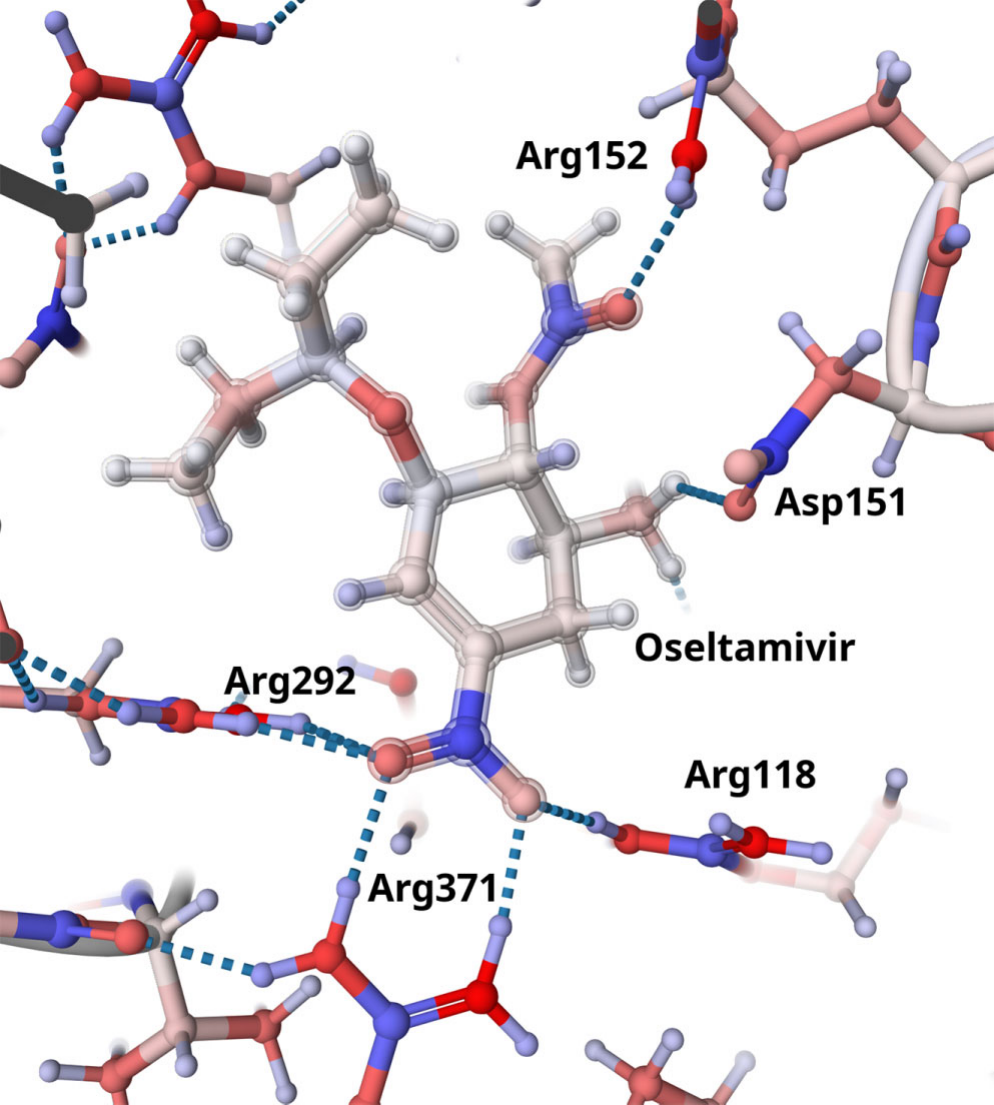
Visualization of partial atomic charges of oseltamivir bonded to neuramidase and corresponding electrostatic interactions, with positive charges depicted in blue and negative charges in red. The most significant electrostatic interaction is between the negatively charged carboxyl group of oseltamivir and the positively charged arginine triad (Arg118, Arg292, and Arg371).

In the second example, we demonstrate the electrostatic interactions between a ligand and a protein. Oseltamivir, an antiviral drug known as Tamiflu, binds to the N1 neuraminidase of the Influenza A virus. We obtained the structure from the PDB with entry ID: 2hu4 [26], added missing hydrogens via Hydride [23], and calculated partial atomic charges using the SQE+qp method [6]. Oseltamivir features a negatively charged carboxylate group that interacts with three surrounding positively charged arginines. Additionally, other functional groups form further electrostatic interactions, strengthening the overall binding affinity between the drug and the protein (see Fig. 3).

## Conclusion

In this article, we presented ACC III, a web application for calculating partial atomic charges using a comprehensive set of empirical methods applicable to diverse molecular systems, including biomacromolecules. Compared to previous versions, ACC III extends the range of available methods, introduces improved visualization via the Mol* viewer, structured mmCIF output with a formally defined extension dictionary, and programmatic access via a web API.

The web interface remains intuitive and platform-independent, enabling easy charge calculation and interactive exploration of results. Computed charges can be downloaded in multiple standard formats (PQR, MOL2, plaintext, and mmCIF). Optional user authentication enables persistent data storage without requiring access.

## Data Availability

Atomic Charge Calculator III is freely available to anyone at https://acc.biodata.ceitec.cz with no login requirement. The source codes of the web server are available at https://github.com/sb-ncbr/AtomicChargeCalculator, and the source codes of the charge computation core are available at https://github.com/sb-ncbr/ChargeFW2, both under the MIT licence. They are also archived on Figshare at https://doi.org/10.6084/m9.figshare.31424435 and https://doi.org/10.6084/m9.figshare.31424393, respectively.

## References

[1] Cho M, Sylvetsky N, Eshafi S et al. The atomic partial charges arboretum: trying to see the forest for the trees. ChemPhysChem. 2020;21:688–96. 10.1002/cphc.202000040 32052532 PMC7317385

[2] Bayly CI, Cieplak P, Cornell W et al. A well-behaved electrostatic potential based method using charge restraints for deriving atomic charges: The RESP model. J Phys Chem. 1993;97:10269–80. 10.1021/j100142a004

[3] Rappe AK, Goddard III WA. Charge equilibration for molecular dynamics simulations. J Phys Chem. 1991;95:3358–63. 10.1021/j100161a070

[4] Cho AE, Guallar V, Berne BJ et al. Importance of accurate charges in molecular docking: quantum mechanical/molecular mechanical (QM/MM) approach. J Comput Chem. 2005;26:915–31. 10.1002/jcc.20222 15841474 PMC3920598

[5] Raček T, Schindler O, Toušek D et al. Atomic Charge Calculator II: web-based tool for the calculation of partial atomic charges. Nucleic Acids Res. 2020;48:W591–6. 10.1093/nar/gkaa367 32402071 PMC7319571

[6] Schindler O, Raček T, Maršavelski A et al. Optimized SQE atomic charges for peptides accessible via a web application. J Cheminform. 2021;13:45. 10.1186/s13321-021-00528-w 34193251 PMC8243439

[7] Holliday JD, Jelfs SP, Willett P et al. Calculation of intersubstituent similarity using R-group descriptors. J Chem Inf Comput Sci. 2003;43:406–11. 10.1021/ci025589v 12653502

[8] Gross KC, Seybold PG, Hadad CM. Comparison of different atomic charge schemes for predicting pKa variations in substituted anilines and phenols. Int J Quant Chem. 2002;90:445–58. 10.1002/qua.10108

[9] Svobodová Vařeková R, Geidl S, Ionescu CM et al. Predicting pK(a) values of substituted phenols from atomic charges: comparison of different quantum mechanical methods and charge distribution schemes. J Chem Inform Model. 2011;51:1795–806. 10.1021/ci200133w 21761919

[10] Reed AE, Weinstock RB, Weinhold F. Natural population analysis. J Phys Chem. 1985;83:735–46. 10.1063/1.449486

[11] Gallegos M, Martín Pendás A. Developing a user-friendly code for the fast estimation of well-behaved real-space partial charges. J Chem Inf Model. 2023;63:4100–14. 10.1021/acs.jcim.3c00597 37339425 PMC10336973

[12] Mortier WJ, Ghosh SK, Shankar S. Electronegativity equalization method for the calculation of atomic charges in molecules. J Am Chem Soc. 1986;108:4315–20. 10.1021/ja00275a013

[13] Mahmoudi S, Gruene T, Schröder C et al. Experimental determination of partial charges with electron diffraction. Nature. 2025;645:88–94. 10.1038/s41586-025-09405-0 40836092 PMC12408337

[14] Ionescu CM, Sehnal D, Falginella FL et al. AtomicChargeCalculator: interactive web-based calculation of atomic charges in large biomolecular complexes and drug-like molecules. J Cheminform. 2015;7:1–13. 10.1186/s13321-015-0099-x 26500704 PMC4613891

[15] Schindler O, Svoboda T, Rošinec A et al. PDBCharges: quantum-mechanical partial atomic charges for PDB structures. Nucleic Acids Res. 2025;53:W457–62. 10.1093/nar/gkaf401 40347106 PMC12230704

[16] Schindler O, Berka K, Cantara A et al. $\alpha$Charges: partial atomic charges for AlphaFold structures in high quality. Nucleic Acids Res. 2023;51:W11–6. 10.1093/nar/gkad349 37158246 PMC10320090

[17] Berman H, Henrick K, Nakamura H. Announcing the worldwide Protein Data Bank. Nat Struct Mol Biol. 2003;10:980. 10.1038/nsb1203-980 14634627

[18] Fleming J, Magana P, Nair S et al. AlphaFold protein structure database and 3D-Beacons: new data and capabilities. J Mol Biol. 2025;437:168967. 10.1016/j.jmb.2025.168967 40133787

[19] Nistor RA, Polihronov JG, Müser MH et al. A generalization of the charge equilibration method for nonmetallic materials. J Chem Phys. 2006;125:094108. 10.1063/1.2346671 16965073

[20] Verstraelen T, Pauwels E, De Proft F et al. Assessment of atomic charge models for gas-phase computations on polypeptides. J Chem Theory Comput. 2012;8:661–76. 10.1021/ct200512e 26596614

[21] Sehnal D, Bittrich S, Deshpande M et al. Mol* Viewer: modern web app for 3D visualization and analysis of large biomolecular structures. Nucleic Acids Res. 2021;49:W431–7. 10.1093/nar/gkab314 33956157 PMC8262734

[22] Linden M, Procházka M, Lappalainen I et al. Common ELIXIR service for researcher authentication and authorisation. F1000Res. 2018;7:ELIXIR–1199. 10.12688/f1000research.15161.1 PMC6124379 30254736

[23] Kunzmann P, Anter JM, Hamacher K. Adding hydrogen atoms to molecular models via fragment superimposition. Algorithms Mol Biol. 2022;17:7. 10.1186/s13015-022-00215-x 35351165 PMC8966362

[24] Dolinsky TJ, Nielsen JE, McCammon JA et al. PDB2PQR: an automated pipeline for the setup of Poisson–Boltzmann electrostatics calculations. Nucleic Acids Res. 2004;32:W665–7. 10.1093/nar/gkh381 15215472 PMC441519

[25] Huang Y, Wang T, Zhong L et al. Molecular architecture of coronavirus double-membrane vesicle pore complex. Nature. 2024;633:224–31. 10.1038/s41586-024-07817-y 39143215 PMC11374677

[26] Russell RJ, Haire LF, Stevens DJ et al. The structure of H5N1 avian influenza neuraminidase suggests new opportunities for drug design. Nature. 2006;443:45–9. 10.1038/nature05114 16915235

